# RARRES2 regulates lipid metabolic reprogramming to mediate the development of brain metastasis in triple negative breast cancer

**DOI:** 10.1186/s40779-023-00470-y

**Published:** 2023-07-25

**Authors:** Yi-Qun Li, Fang-Zhou Sun, Chun-Xiao Li, Hong-Nan Mo, Yan-Tong Zhou, Dan Lv, Jing-Tong Zhai, Hai-Li Qian, Fei Ma

**Affiliations:** 1 Department of Medical Oncology, National Cancer Center/National Clinical Research Center for Cancer/Cancer Hospital, Chinese Academy of Medical Sciences and Peking Union Medical College, Beijing, 100021 China; 2 State Key Laboratory of Molecular Oncology, National Cancer Center/National Clinical Research Center for Cancer/Cancer Hospital, Chinese Academy of Medical Sciences and Peking Union Medical College, Beijing, 100021 China

**Keywords:** RARRES2, Lipid metabolic reprogramming, Brain metastasis (BrM), Breast cancer

## Abstract

**Background:**

Triple negative breast cancer (TNBC), the most aggressive subtype of breast cancer, is characterized by a high incidence of brain metastasis (BrM) and a poor prognosis. As the most lethal form of breast cancer, BrM remains a major clinical challenge due to its rising incidence and lack of effective treatment strategies. Recent evidence suggested a potential role of lipid metabolic reprogramming in breast cancer brain metastasis (BCBrM), but the underlying mechanisms are far from being fully elucidated.

**Methods:**

Through analysis of BCBrM transcriptome data from mice and patients, and immunohistochemical validation on patient tissues, we identified and verified the specific down-regulation of retinoic acid receptor responder 2 (RARRES2), a multifunctional adipokine and chemokine, in BrM of TNBC. We investigated the effect of aberrant RARRES2 expression of BrM in both in vitro and in vivo studies. Key signaling pathway components were evaluated using multi-omics approaches. Lipidomics were performed to elucidate the regulation of lipid metabolic reprogramming of RARRES2.

**Results:**

We found that down-regulation of RARRES2 is specifically associated with BCBrM, and that RARRES2 deficiency promoted BCBrM through lipid metabolic reprogramming. Mechanistically, reduced expression of RARRES2 in brain metastatic potential TNBC cells resulted in increased levels of glycerophospholipid and decreased levels of triacylglycerols by regulating phosphatase and tensin homologue (PTEN)-mammalian target of rapamycin (mTOR)-sterol regulatory element-binding protein 1 (SREBP1) signaling pathway to facilitate the survival of breast cancer cells in the unique brain microenvironment.

**Conclusions:**

Our work uncovers an essential role of RARRES2 in linking lipid metabolic reprogramming and the development of BrM. RARRES2-dependent metabolic functions may serve as potential biomarkers or therapeutic targets for BCBrM.

**Supplementary Information:**

The online version contains supplementary material available at 10.1186/s40779-023-00470-y.

## Background

Among women worldwide, breast cancer is the most common cancer and the second leading cause of cancer-related death [1]. Brain metastasis (BrM), the most lethal form of breast cancer, represents an important cause of morbidity and mortality, with one-year survival of approximately 20% [2]. In the case of triple-negative breast cancer (TNBC), which is the most aggressive subtype, as many as 46% of patients suffer metastasis to the brain, after which their median survival is only 4.9 months [3]. Despite the advances in systemic treatment in recent years, treating breast cancer brain metastasis (BCBrM) is challenging and generally ineffective [4, 5]. Therefore, there is an urgent need to improve our understanding of the underlying mechanisms of BCBrM, particularly in the TNBC subtype.

Cancer cells display significant metabolic reprogramming to support metastatic competence [6], one of which is lipid metabolic reprogramming [7]. Recent evidence has suggested that breast cancer cells capable of metastasizing to the brain presented with unique lipid metabolic signatures, and perturbation of lipid metabolism in these cells inhibited BrM [8, 9]. Similarly, brain-tropic melanoma cancer cells have been reported to take advantage of the high-fat microenvironment of the brain to facilitate their metastatic outgrowth [10]. These limited findings shed light on the potential importance of lipid metabolic reprogramming in BrM, but the precise underlying mechanisms are far from been fully elucidated.

Retinoic acid receptor responder 2 (RARRES2), also known as tazarotene-induced gene 2 or chemerin, is an important adipokine which regulates adipogenesis and adipocyte lipid metabolism [11, 12]. Several studies have reported a tumor suppressive role of RARRES2 in cancer development [13–15], however, not much is currently known about the functions of RARRES2 in cancer cell lipid metabolism, and nothing is known regarding the association with RARRES2, lipid metabolism, and BCBrM.

Herein, we for the first time identified RARRES2 as the most significantly downregulated gene in BCBrM compared with primary breast tumors. We hypothesized and verified that the decreased expression of RARRES2 might promote BCBrM through regulating lipid metabolic reprogramming. Mechanistically, reduced expression of RARRES2 in TNBC cells increased glycerophospholipid levels and decreased levels of triacylglycerols (TAGs) by regulating the phosphatase and tensin homologue (PTEN)-mammalian target of rapamycin (mTOR)-sterol regulatory element-binding protein 1 (SREBP1) signaling pathway to facilitate the colonization of breast cancer cells in adapting to the brain microenvironment. Collectively, we provide a pathogenic mechanism linking RARRES2 and lipid metabolic reprogramming in the development of BrM in breast cancer, with the anticipation that the RARRES2-dependent metabolic functions may serve as biomarkers or therapeutic targets for BCBrM in TNBC.

## Methods

### Tumor samples from patients

Biopsies of primary breast tumors and breast tumors that had metastasized to the brain were obtained from patients with TNBC at the National Cancer Center/National Clinical Research Center for Cancer/Cancer Hospital of the Chinese Academy of Medical Sciences and Peking Union Medical College. Single-cell RNA sequencing (scRNA-seq) involved the collection of 1 BrM sample from patients with primary TNBC, while immunohistochemistry (IHC) employed 3 BrM samples and 3 primary breast tumor tissues from patients with primary TNBC. This study was approved by the local institutional review board (NCC2020C-503).

### Preparation of single cell suspension

BrM tumor tissues were initially stored on ice in RNAlater stabilization reagent (Qiagen, Germany), then resuspended in RPMI-1640 (Gibco, USA) and digested gently using the MACS Tumor Dissociation Kit (Miltenyi Biotec, USA) for 60 min on a rotor at 37 °C. Dissociated cells were filtered through a 40 μm cell strainer (Merck, Germany) and subjected to red blood cell lysis (Sangon Biotech, China). Pelleted cells were washed twice with phosphate-buffered saline (PBS, Invitrogen, USA) by centrifugation at 400 g for 10 min and resuspended in PBS containing 1% fetal bovine serum (FBS, HyClone, USA).

### scRNA-seq and data preprocessing

scRNA-seq libraries were prepared using the Chromium Next GEM Single Cell 3ʹ Kit v3.1 (10 × Genomics) according to the manufacturer’s instructions. Single-cell RNA libraries were purified, assessed for quality, and sequenced with 150 bp paired-end reads using an Illumina HiSeq X-Ten sequencer. The FASTQ sequence files were mapped to the human reference genome GRCh38 using Cell Ranger Count (version 3.0.2).

Single-cell transcriptomic sequences were pre-processed using the Seurat pipeline [16–18]. During the quality control (QC), cells with detected genes less than 500 or more than 9000, and mitochondrial content higher than 10% were excluded from further analyses. Since we only collected one TNBC BrM sample, in order to separate tumor cells and stromal cells, we merged this sample (BrM1) with a TNBC BrM sample (BrM2) from the public dataset GSE186344 of the Gene Expression Omnibus (https://www.ncbi.nlm.nih.gov/geo/) (Additional file 1: Fig. S1a, b). The count matrix of cells that passed the QC was then normalized using global scaling log normalization followed by principal component analysis on the variable genes selected by mean variability plot method in FindVariableFeatures, and batch effects were removed using the “harmony” package. For clustering, a uniform manifold approximation and projection dimensional reduction was performed on the scaled matrix using the first 18 principal component analysis components and a FindClusters function was set at a resolution of 0.5.

### Identification of cluster-specific genes and marker-based classification

To identify marker genes, the FindAllMarkers function was used with likelihood-ratio test for single cell gene expression. For each cluster, only genes that were expressed in more than 25% of cells with at least 0.25-fold change were included. Cells were annotated on the basis of the expression of marker genes: endothelial cells, based on the marker genes *CLDN5* and *PECAM1*; mural cells, based on marker genes *RGS5* and *HIGD1B*; mesenchymal cells, based on marker genes *ISLR* and *CTHRC1*; cancer epithelial cells, based on marker genes *EPCAM, KRT15* and *KRT17*; T cells, based on marker genes *CD3D* and *CD2*; B cells, based on marker genes *JCHAIN* and *MZB1*; macrophages and monocytes, based on marker genes *LYZ, APOE* and *S100A8*; dendritic cells, based on marker genes *CD1C, HLA-DQA1* and *HLA-DRA*; and astrocytes, based on marker genes *S100B* and *MT3* (Additional file 1: Fig. S1c).

### Scoring gene sets of primary breast tumor and BrM with irGSEA

Cells annotated as cancer epithelial cells were extracted from the BrM matrix (BrM) and integrated with cancer epithelial cells from 8 primary breast tumor tissues from patients (Primary) with TNBC derived from GSE176078 (Additional file 1: Fig. S1d), and the batch effects were removed using the “harmony” package. Sets of genes in cancer epithelial cells from primary breast cancer tumors (Primary) and breast cancer tumors that had metastasized to the brain (BrM) were compared using the singscore algorithm in irGSEA (https://github.com/chuiqin/irGSEA).

### Identification of genes differentially expressed between primary TNBC and BrM

Microarray dataset GSE12237 and GSE19184 were analyzed by GEO2R (https://www.ncbi.nlm.nih.gov/geo/geo2r/) to analyze genes that are downregulated in BrM relative to primary breast cancer cells. Raw count data from RNA sequencing of patients with paired primary breast cancer tumors and metastatic tumors in the brain were downloaded from https://github.com/npriedig/jnci_2018. Expression of RARRES2 was compared between the two types of matched tumors using a two-tailed paired *t* test. To further analyze the expression of RARRES2, we analyzed the expression of RARRES2 (probe id: 209496_at) between metastatic brain tissue samples and metastatic samples from other organs (bone, lung, and liver) in GSE100534, GSE14020 and GSE62598.

### Identification of RARRES2 co-expression genes

We used the online platform cBioPortal (https://www.cbioportal.org/) to extract RARRES2 co-expression genes within the TCGA breast cancer transcriptome sequencing data set. Genes with Pearson correlation coefficient > 0.3 or <  − 0.3 and *P* < 0.5 were selected.

### Bioinformatic assay for gene function enrichment

To analyze the potential functions of gene sets, DAVID Bioinformatics Resource 6.8 (https://david.ncifcrf.gov/) and Gene-set enrichment analysis (https://www.gsea-msigdb.org/gsea) were used for enrichment analysis. Degree of enrichment was defined using Fisher’s exact test, and gene set enrichment analysis was performed using gene set permutation. Results were ranked based on the signal-to-noise ratio.

### IHC staining of tumor tissues from patients

The formalin fixation and paraffin embedding tissue sections were dried overnight at 60 ℃, deparaffinized in xylene for 30 min, rehydrated through a gradient alcohol solution, boiled for 15 min in antigen retrieval solution containing EDTA (ZLI-9066, Zsgb-Bio, China), treated for 10 min with endogenous peroxidase blocker (PV-9000, Zsgb-Bio, China), and incubated for 10 min with normal goat serum (ZLI-9022, Zsgb-Bio, China). The sections were incubated overnight at 4 ℃ with a rabbit monoclonal primary antibody against RARRES2 (10216-1-AP, Proteintech, China), then incubated for 25 min with biotinylated secondary antibodies (PV-9000, Zsgb-Bio, China). Color development was performed with 3,3N-diaminobenzidine tertrahydrochloride (ZLI-9019, Zsgb-Bio, China). The sections were counterstained with hematoxylin, mounted by slides with neutral balsam, and scanned by Aperio patholgy scanner (Leica, Germany).

### Mouse model of breast cancer metastasis to the brain

All animal experiments were approved by the Animal Control Committee of the National Cancer Center/National Clinical Research Center for Cancer/Cancer Hospital of the Chinese Academy of Medical Sciences and Peking Union Medical College (NCC2022A116). Female BALB/c (*n* = 12) and BALB/c-Nu (6 weeks old, *n *= 11) were purchased from Beijing Vital River Laboratory Animal Technology (Beijing, China), housed on a 12 h light/dark cycle and given free access to normal food and water. Mice were randomly assigned to experimental groups for all the experiments.

For intracranial inoculations, following published protocol [19], the 6-week-old BALB/c mice (*n* = 6 for each group) were anesthetized with tribromoethanol and injected with 3 × 10^4^ 4T1 cells expressing luciferase in 5 μl PBS into the brain at the rate of 2.5 μl of cell suspension per minute at 2 mm to the right of bregma and 1 mm anterior to the coronal suture. For intracardiac injections, 1 × 10^5^ 4T1 cells expressing luciferase were diluted in 100 μl PBS and were injected into the left ventricle of the hearts of 6-week-old BALB/c-Nu mice (*n* = 5 for Control, *n* = 6 for RARRES2-OE).

For bioluminescence imaging (BLI), mice were given the substrate D-luciferin potassium (150 mg/kg, HY-12591B, MedChemExpress, USA) by intraperitoneal injection and then subjected to IVIS LUMINA XRMS (PerkinElmer, USA) to monitor the growth of tumor cells. At the end of the experiments, brain was collected for examination of metastasis.

### Cell culture

The MDA-MB-231, 4T1 and 293 TN cell lines were obtained from the National Infrastructure of Cell Line Resources (Beijing, China). MDA-MB-231 and 293 TN cells were cultured in DMEM (Gibco, USA) supplemented with 10% FBS, and 4T1 cells were maintained in RPMI 1640 (Gibco, USA) supplemented with 10% FBS. All cell cultures were incubated at 37 °C in a humidified incubator containing 5% CO_2_.

### Plasmid constructs and lentivirus transduction

The full-length coding sequences for human RARRES2 (NM_002889) and mouse RARRES2 (NM_027852) were cloned separately into the vector pCDH-EF1-copGFP-T2A-Puro (Addgene plasmid # 72,263). Each of the following short hairpin RNAs was cloned separately into the vector PLKO.1-TRC (Addgene plasmid #10,878). human-shRARRES2-1: ACCGGTGCCCTTCCCAGCTGGAATATTCTCGAGAATATTCCAGCTGGGAAGGGCTTTTTTGAATTC; human-shRARRES2-2: ACCGGTGCTTCTACTTCCCTGGACAGTCTCGAGACTGTCCAGGGAAGTAGAAGCTTTTTTGAATTC; mice-shRARRES2-1: ACCGGTAGCCGGAGTGCACAATCAAACCTCGAGGTTTGATTGTGCACTCCGGCTTTTTTGAATTC; or mice-shRARRES2-2: ACCGGTGCACAATCAAACCAAACGGGACTCGAGTCCCGTTTGGTTTGATTGTGCTTTTTTGAATTC. All plasmids were verified by DNA sequencing.

Each of these plasmids was co-transfected with the packaging vectors psPAX2 and pMD2.G (Addgene, USA) into 293TN cells using Lipofectamine 2000 (Invitrogen, USA). Culture medium containing lentivirus was collected at 48 h and 72 h after transfection, filtered through hydrophilic polyethersulfone membrane with 0.45 μm pores (SLHPR33RB, Millipore, USA), and used to infect MDA-MB-231 or 4T1 cells for 48 h in the presence of 4 μg/ml polybrene (HY-112735, MedChemExpress, USA). Stably transduced cell lines were selected using puromycin (A610593-0025, Sangon Biotech, China).

### siRNA transfection

RARRES2 overexpression (RARRES2-OE) and its control MDA-MB-231 cells were seeded into 6-well plates (60% confluence cells/well), incubated for 24 h, then transfected with the following siRNAs (100 pmol/well) using Lipofectamine 2000 respectively. The RNA was extracted at 48 h post-transfection and the total protein lysates were extracted at 72 h. The control siNC is a scrambled nonspecific sequence used as a negative control. The sequence of siNC and siCMKLR1 are listed below. siNC: 5ʹ-UUCUCCGAACGUGUCACGUTT-3ʹ (sense), 5ʹ-ACGUGACACGUUCGGAGAATT-3ʹ (antisense); siCMKLR1-1: 5ʹ-GCAAUGGUCUGGUGAUCAUTT-3ʹ (sense), 5ʹ-AUGAUCACCAGACCAUUGCTT-3ʹ (antisense);siCMKLR1-2: 5ʹ-CCAUGUGCAAGAUCAGCAATT-3ʹ (sense), 5ʹ-UUGCUGAUCUUGCACAUGGTT-3ʹ (antisense); siCMKLR1-3: 5ʹ-CCUCACCAUCGUGUGCAAATT-3ʹ (sense), 5ʹ-UUUGCACACGAUGGUGAGGTT-3ʹ (antisense).

### RNA extraction and RNA sequencing

Total RNA from MDA-MB-231 RARRES2-OE and control cells was prepared using the TRIzol reagent (Invitrogen, USA). RNA sequencing was performed on HiSeq X Ten sequencer (Illumina, USA), paired-end 150 bp run. The number of reads aligning to each transcript were counted with hisat2 and SAM tools. Differentially expressed genes were calculated by DEseq2. Unsupervised clustering and Gene Ontology analyses of differentially expressed genes were performed on DAVID (https://david.ncifcrf.gov).

### Real-time quantitative polymerase chain reaction (RT-qPCR)

Total RNA of the above cultured MDA-MB-231 cells was isolated using TRIzol regent (Invitrogen, USA). And then 1 mg of RNA was treated with genomic DNase and retro-transcripted using PrimeScript RT reagent Kit (RR037A, Takara, China). qPCR was performed in triplicate using 25 ng of cDNA with validated qPCR primers, TB Green Premix Ex Taq II Kit (RR820A, Takara, China), and Applied Biosystems Real-time PCR System (QSDX, Thermo Fisher, USA). The reaction was performed according to the manufacturer’s protocol using QSDX Real-time PCR System. Data were analyzed following the 2^−∆∆Ct^ method. The Ct values of the genes of interest were normalized to the Ct value of the housekeeping control, GAPDH. The primers used for qPCR are listed below. Acetyl-CoA carboxylase alpha (ACACA): forward 5ʹ-ATGTCTGGCTTGCACCTAGTA-3ʹ, reverse 5ʹ-CCCCAAAGCGAGTAACAAATTCT-3ʹ; hydroxymethylglutaryl-CoA reductase (HMGCR): forward 5ʹ-TGATTGACCTTTCCAGAGCAAG-3ʹ, reverse 5ʹ-CTAAAATTGCCATTCCACGAGC-3ʹ; RARRES2: forward 5ʹ-AGAAACCCGAGTGCAAAGTCA-3ʹ, reverse 5ʹ-AGAACTTGGGTCTCTATGGGG-3ʹ; chemokine-like receptor-1 (CMKLR1): forward 5ʹ-GCCAACCTGCATGGGAAAATA-3ʹ, reverse 5ʹ-GTGAGGTAGCAAGCTGTGATG-3ʹ; fatty acid synthase (FASN): forward 5ʹ- AAGGACCTGTCTAGGTTTGATGC-3ʹ, reverse 5ʹ-TGGCTTCATAGGTGACTTCCA-3ʹ.

### Western blotting

Total protein in extracts from the above breast cancer cell cultures was estimated using Coomassie brilliant blue, and equal amounts (40 μg) were fractionated by 10% sodium dodecyl sulfate–polyacrylamide gel electrophoresis, and proteins were transferred to PVDF membranes (IPVH00010, Millipore, USA), which were blocked for 1 h in PBS containing 5% non-fat dry milk, then incubated overnight at 4 °C with primary antibodies against the following proteins. The following antibodies were obtained from Cell Signaling Technology and used at a 1:1000 dilution: mTOR (catalog No. 2972), phospho-Ser2448 mTOR (catalog No. 2971), GAPDH (catalog No. 5174), pan-Akt (catalog No. 4691), phospho-Ser473 Akt (catalog No. 4060), and PTEN (catalog No. 9188). Mouse monoclonal antibodies were used against SREBP1 (ab3259, 5 µg/ml, Abcam, UK) and against β-actin (A5316, 1:5000, Sigma, USA). Polyclonal antibody against RARRES2 was purchased from Proteintech (10216-1-AP) and used at 1:1000 dilution.

On the next day, the membranes were incubated for 1 h at room temperature with horseradish peroxidase-conjugated secondary antibody (ZB-2301 and ZB-2305, Zsgb-Bio, Cjina). Antibody binding was visualized using Super ECL Plus (P1010, Applygen, China) or SuperSignal™ West Femto Maximum Sensitivity Substrate (34,095, Thermo Fisher, USA) and Image800 fluorescence imaging system (Cytiva, USA).

### Cell proliferation assay

The above breast cancer cells were measured for their proliferation by the xCELLigence Real-time Cell Analysis (RTCA) assay (ACEA Biosciences, USA) or IncuCyte S3. Cells (8.0 × 10^3^ cells in 150 μl medium/well) were seeded in 16-well E-plates or 96-well plates, and the cell index was recorded every 1 h and continued for 72 h until the cell growth reached the plateau stage. IncuCyte S3 image analysis software was set to detect the edges of the cells and to determine their confluence in percentage. The cell index was normalized to the cell index at 0 h (normalized cell index).

### In vitro invasion assay

To evaluate the invasion capacity of the cells, the above breast cancer cells in serum-free growth medium (1.0 × 10^5^ per well) were seeded into the top chamber of a 24-well Transwell plate containing a layer of 0.5% Matrigel (catalog No. 3422, Corning, USA). The bottom chamber was filled with 600 μl DMEM containing 20% FBS. The Transwell plates were incubated at 37 °C in a humidified incubator containing 5% CO_2_ for 24 h, then cells in the chambers were fixed in methanol for 15 min, then stained with 0.5% crystal violet for 15 min. Cells that did not penetrate the membrane were removed using a cotton swab, and those that passed through the membrane were photographed. Five random microscopic fields were selected, and the average area occupied by cells was calculated with ImageJ software.

### Identification of RARRES2-related lipids by mass spectrometry

shRARRES2-2 and control MDA-MB-231 cells were collected in 1.5 ml centrifuge tubes, 6 technical replicates per group, 8.0 × 10^6^ cells per sample. Then, 1 ml precooled extractant (70% methanol aqueous solution) was added to each sample and vortexed for 1 min. The mixture was frozen in liquid nitrogen for 3 min and agitated the cells 3 times for 2 min each, and centrifuged at 12,000 rpm and 4 °C for 10 min. The supernatants were transferred to sample bottles for LC-MS/MS. The QC sample was prepared by mixing an equal aliquot of 20 μl supernatants from all of the samples. LC-MS/MS analyses were performed using an UHPLC system (1290, Agilent Technologies, USA), equipped with a Kinetex C18 column (2.1 × 100 mm, 1.7 μm, Phenomen, USA). The QE mass spectrometer was used to acquire MS/MS spectra on data dependent acquisition mode using the control of the acquisition software (Xcalibur 4.0.27, Thermo, USA).

### Statistical analysis

Data were reported as mean ± standard deviation of at least three independent experiments. Unpaired Student’s *t* test and the Wilcoxon rank-sum test were used to compare the continuous variables between two groups with or without normally distributed variables, respectively. If there were more than two groups, one-way ANOVA was used. For the proliferation assay data, two-way ANOVA was used. The correlation between variables was measured using Pearson’s or Spearman’s correlation coefficient. Details of statistical tests used are indicated in the respective figure legend. The differences between conditions were assessed for their significance in GraphPad Prism 8.0 using the statistical tests indicated in the corresponding figure legends. A two-sided *P* value of less than 0.05 was deemed to indicate statistical significance.

## Results

### RARRES2 expression is decreased in BCBrM

**Fig. 1:**
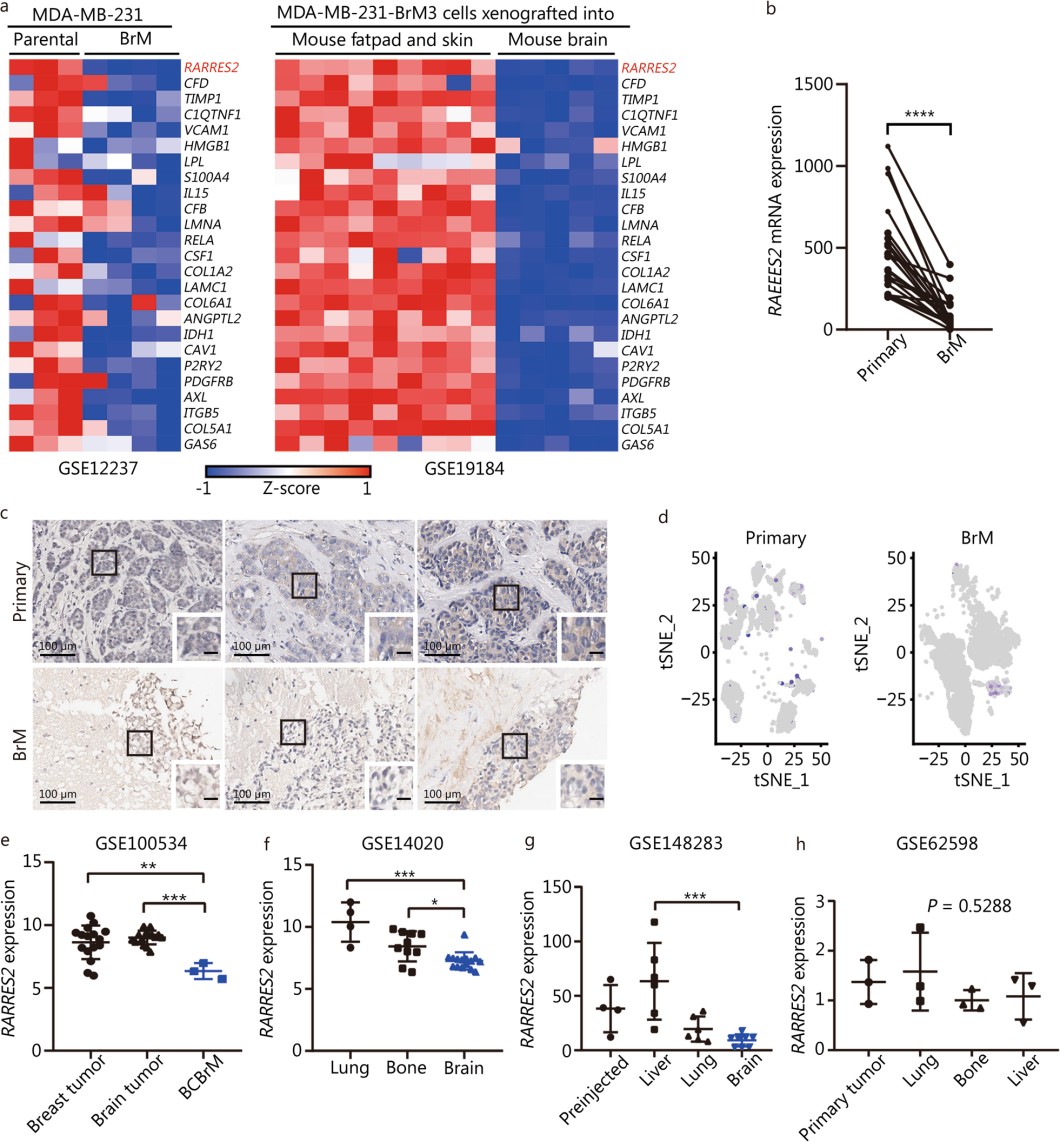
RARRES2 is downregulated in breast cancer brain metastasis (BrM). **a** Heatmap showing transcription levels of 25 common adipokine-related genes in parental and MDA-MB-231 BrM cells (left), and in MDA-MB-231-BrM3 cells xenografted into mouse fatpad and skin or brain (right). Data came from the public datasets indicated at the bottom of the heatmaps. Blue indicates lower expression, while red indicates higher expression. **b** Comparison of RARRES2 expression between paired primary breast tumors (*n* = 22) and metastatic tumors to the brain (*n* = 22). ^****^P < 0.0001 (paired-sample *t* test).** c** Immunohistochemistry staining for RARRES2 in formalin-fixed, paraffin-embedded sections from primary triple negative breast cancer (TNBC, upper row) or tumors that had metastasized to the brain (lower row) in our patient. The black boxes indicate the area shown at higher magnification (Scale bar = 10 μm) in the insets at the lower right of each micrograph (Scale bar = 100 μm). **d** scRNA-seq data analysis of RARRES2 expression from human TNBC (Primary) and BrM cancer epithelial cells, presented as tSNE plots. **e** RARRES2 expression in human breast tumor tissues (*n* = 16), brain tumor tissues (*n* = 16), and BCBrM tissues (*n* = 3). Data were from the public dataset GSE100534. ^**^P = 0.0016, ^***^P = 0.0003 (one-way ANOVA followed by Dunnett’s multiple-comparisons test). **f** RARRES2 expression in human breast cancer that had metastasized to lung (*n* = 4), bone (*n* = 10) or brain (*n* = 15). Data were from the public dataset GSE14020. ^*^P = 0.019, ^***^P < 0.001 (one-way ANOVA followed by Dunnett’s multiple-comparisons test). **g** RARRES2 expression in breast cancer cells before injection (*n* = 4) into mice and after metastasis to the liver (*n* = 6), lung (*n* = 6), or brain (*n* = 8). Data came from GSE148283. ^***^P = 0.0003 (one-way ANOVA followed by Dunnett’s multiple-comparisons test). **h** RARRES2 expression in primary breast tumors (*n* = 3) and tumors that had metastasized to the lung (*n* = 3), bone (*n* = 3) or liver (*n* = 3) in mice. Data came from the public dataset GSE62598. The *P-*value was determined using one-way ANOVA. RARRES2 retinoic acid receptor responder 2, BCBrM breast cancer brain metastasis, scRNA-seq single-cell RNA-sequencing

To investigate the potential lipid metabolic reprogramming signature of BrM, we employed scRNA-seq analysis on TNBC primary tumors and BrM samples. The two BrM datasets used in this study were obtained from our own scRNA-seq data (BrM1) and GSE186344 (BrM2) (Additional file 1: Fig. S1a). By assessing the expression levels of specific marker genes, a total of 9 distinct cell clusters were identified (Additional file 1: Fig. S1b, c). Through a comparative analysis of tumor cell clusters from primary tumors (*n* = 87,045 tumor cells, GSE176078) and BrM (*n* = 29,173 tumor cells) (Additional file 1: Fig. S1d), we discovered that BrM tumor cells exhibited an upregulated lipid synthesis and fatty acid metabolism signatures, which were consistent with previous literature (Additional file 1: Fig. S1e). In order to identify a potential adipokine that might be of critical importance in the initiation and development of BCBrM, we conducted data mining analysis utilizing Gene Expression Omnibus public datasets. Specifically, the expression of 823 genes encoding adipokines or key enzymes in lipid metabolism (annotated with adipokine in Genecard) were compared between human TNBC MDA-MB-231 parental cells and MDA-MB-231 BrM (GSE12237), as well as between MDA-MB-231-BrM cells xenografted into mouse fatpad and skin, and brain (GSE19184) (Fig. 1a). Significant genes that were consistently downregulated in BrM compared with parental cells or tumor cells injected into the fatpad across both datasets were identified (*P* < 0.05). Notably, RARRES2, also known as the adipokine chemerin, emerged as the most significantly downregulated gene in BCBrM (Fig. 1a). To validate our findings, we conducted an additional analysis using an independent patient cohort consisting of paired primary breast tumors and BrM [20]. The expression of RARRES2 was examined in this cohort, revealing a significantly lower level of RARRES2 expression in BrM compared to matched primary breast tumor samples (*P *< 0.0001, Fig. 1b). Furthermore, we performed IHC to assess the expression of RARRES2 protein in human tumor samples. The results demonstrated a notable decrease in RARRES2 expression in BrM samples when compared to primary breast tumors (Fig. 1c). Additionally, based on the scRNA-seq data mentioned above, we observed lower levels of RARRES2 expression in BrM compared to primary breast cancer (Fig. 1d).

To further investigate the expression of RARRES2 in different tumor sites, we analyzed another separate patient cohort comprising unmatched primary breast tumors and brain metastasized lesions. In this cohort, RARRES2 expression was found to be significantly decreased in BCBrM compared with primary breast tumors, and moreover, with primary brain tumors (GSE100534) (*P* = 0.0016, *P* = 0.0003, Fig. 1e). Analysis of GSE14020 revealed that the level of RARRES2 was notably lower in the breast cancer that metastasized to brain than that to bone and lung (*P* = 0.019, *P *< 0.001, Fig. 1f). Additionally, we then examined the expression of RARRES2 using the MetMap data (GSE148283) and found a significant downregulation of RARRES2 in the breast cancer cells metastasized to the brain compared to metastasis in the liver (*P* = 0.0003, Fig. 1g). Furthermore, when analyzing the GSE62598 dataset, we did not observe a decrease in RARRES2 expression in liver, bone, or lung metastases compared to primary breast tumors (Fig. 1h). Collectively, these findings suggest that the expression of RARRES2 is decreased specifically in BrM of breast cancer, highlighting a potential association between RARRES2 downregulation and BrM.

### RARRES2 depletion promotes BrM formation

**Fig. 2:**
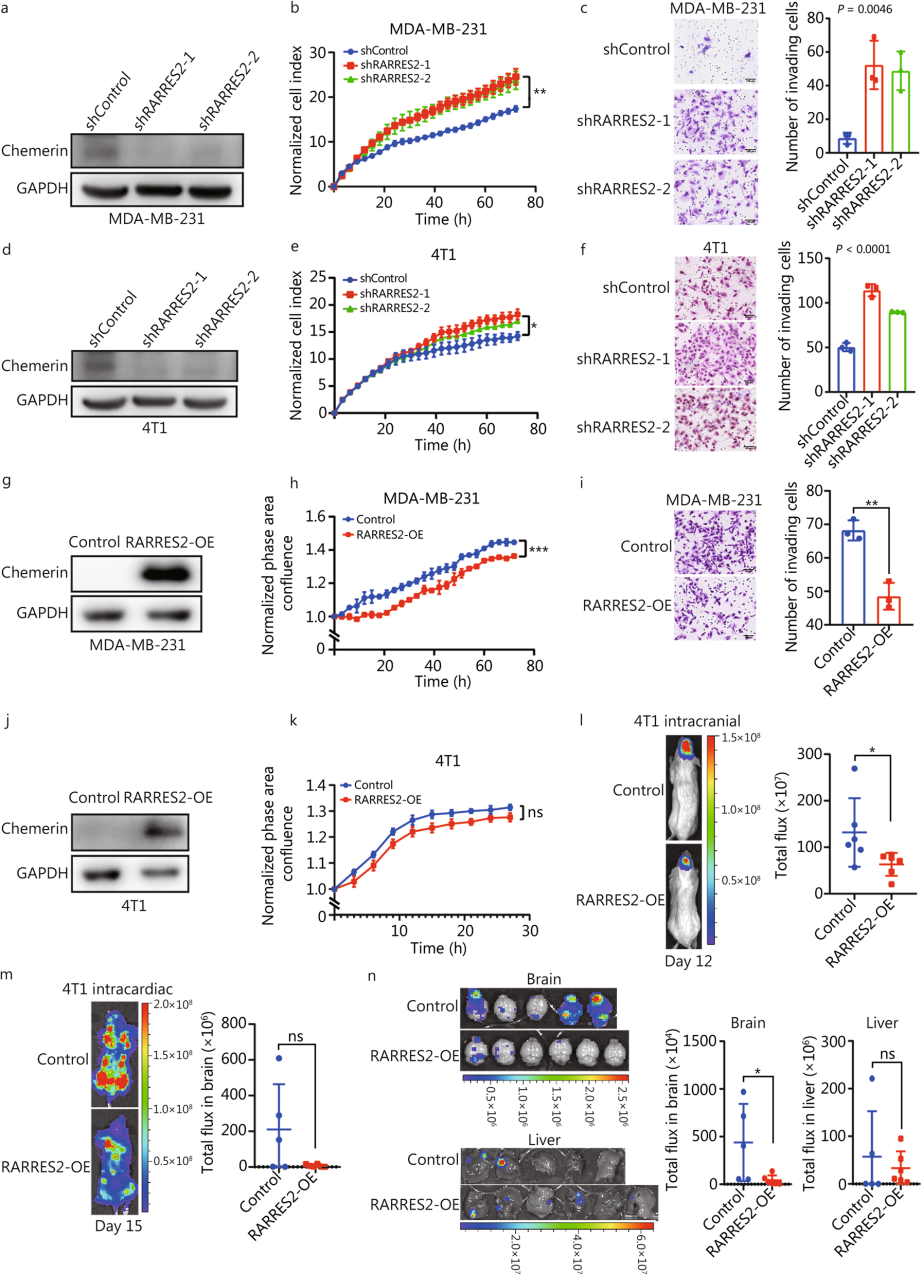
RARRES2-OE inhibits metastasis of breast cancer to the brain. **a–c** MDA-MB-231 cells were stably transduced with lentivirus encoding short hairpin RNAs targeting RARRES2 (shRARRES2-1 and -2) or negative control RNA (shControl). Then, the cells were assayed for RARRES2 expression (**a**), proliferation (**b**) and invasion (**c**). Significance was assessed in panel (**b**) using two-way ANOVA with Geisser-Greenhouse correction (^**^P = 0.0023) or in panel **c** using one-way ANOVA followed by Dunnett’s multiple-comparisons test. **d-f** The experiments in panels **a–c** were repeated using cultures of 4T1 cells. In panel **e**, ^*^P = 0.0177. **g–i** Immunoblot of lysate confirming overexpression of RARRES2 (**g**), cell proliferation assay (**h**) and invasion assay (**i**) from MDA-MB-231 cells infected with control or encoding RARRES2 lentivirus (RARRES2-OE). Two-way ANOVA with Geisser-Greenhouse correction (**h**), ^***^P = 0.0005. Two-tailed unpaired sample *t*-test (**i**), ^**^P = 0.0025. **j, k** Same as **g, h** for 4T1 cells, *P* = 0.1053 (**k**). **l** Bioluminescence imaging (BLI) at 12 d after intracranial injection of RARRES2-OE and Control 4T1 cells. The dots on the right represent the photon flux in brain for each mouse at day 12 (*n* = 6 for each group). ^*^P = 0.0303 (two-sided Mann–Whitney test). **m** BLI at 15 d after intracardiac injection of RARRES2-OE and Control 4T1 cells. The dots on the right represent the photon flux in brain for each mouse at day 15 (*n* = 5 for Control, *n* = 6 for RARRES2-OE). Two-tailed unpaired-samples *t* test. **n** BLI of brain and liver from mice receiving intracardiac injection in the experiments. The dots on the right represent the photon flux in brain or liver for each mouse at day 15 (*n* = 5 for Control, *n* = 6 for RARRES2-OE). ^*^P = 0.0401 (two-tailed unpaired-samples *t* test). RARRES2 retinoic acid receptor responder 2, RARRES2-OE RARRES2 overexpression, ns non-significant

To explore the role of RARRES2 in BrM of TNBC, our next objective was to investigate whether RARRES2 expression could influence the invasiveness or proliferation of brain metastatic cell. Initial analysis of the Genotype-Tissue Expression database revealed relatively low levels of RARRES2 expression in normal brain tissue (Additional file 1: Fig. S2). This led us to speculate that the decreased expression of RARRES2 in breast cancer cells that have metastasized to the brain may be an adaptation to the brain microenvironment. To simulate the low expression of RARRES2 in BrM, two distinct short hairpin RNAs were employed to stably knockdown RARRES2 (RARRES2-KD) in MDA-MB-231 and 4T1 cells, respectively (Fig. 2a, d). Subsequently, it was observed that RARRES2-KD significantly promoted cell proliferation and invasion in both cell lines (*P* = 0.0023, *P* = 0.0177, Fig. 2b, c, e, f). Conversely, overexpression of RARRES2 (RARRES2-OE) (Fig. 2g, j) significantly inhibited cell proliferation and invasion of MDA-MB-231 cells (*P* = 0.0005, *P* = 0.0025, Fig. 2h, i).

In order to track tumor cell behavior in vivo, we utilized BLI of luciferase-labeled RARRES2-OE 4T1 cells. Remarkably, RARRES2-OE demonstrated a significant inhibition of 4T1 cells growth when injected intracranially into the brains of mice (*P* = 0.0303, Fig. 2l), although there was no statistical difference in vitro (Fig. 2k). Furthermore, RARRES2-OE also suppressed the metastasis of 4T1 cells to the brain following intracardiac injection (*P* = 0.0401, Fig. 2m, n). However, it is noteworthy that RARRES2-OE did not exhibit a significant inhibition of liver metastases (Fig. 2n). Taken together, these findings strongly suggest that RARRES2 plays a crucial role in BCBrM, as high levels of RARRES2 impede the process of BrM in breast cancer.

### Functional enrichment analyses indicate RARRES2 regulates lipid metabolic reprogramming and phosphatidylinositol 3-kinase (PI3K) signaling pathway

**Fig. 3:**
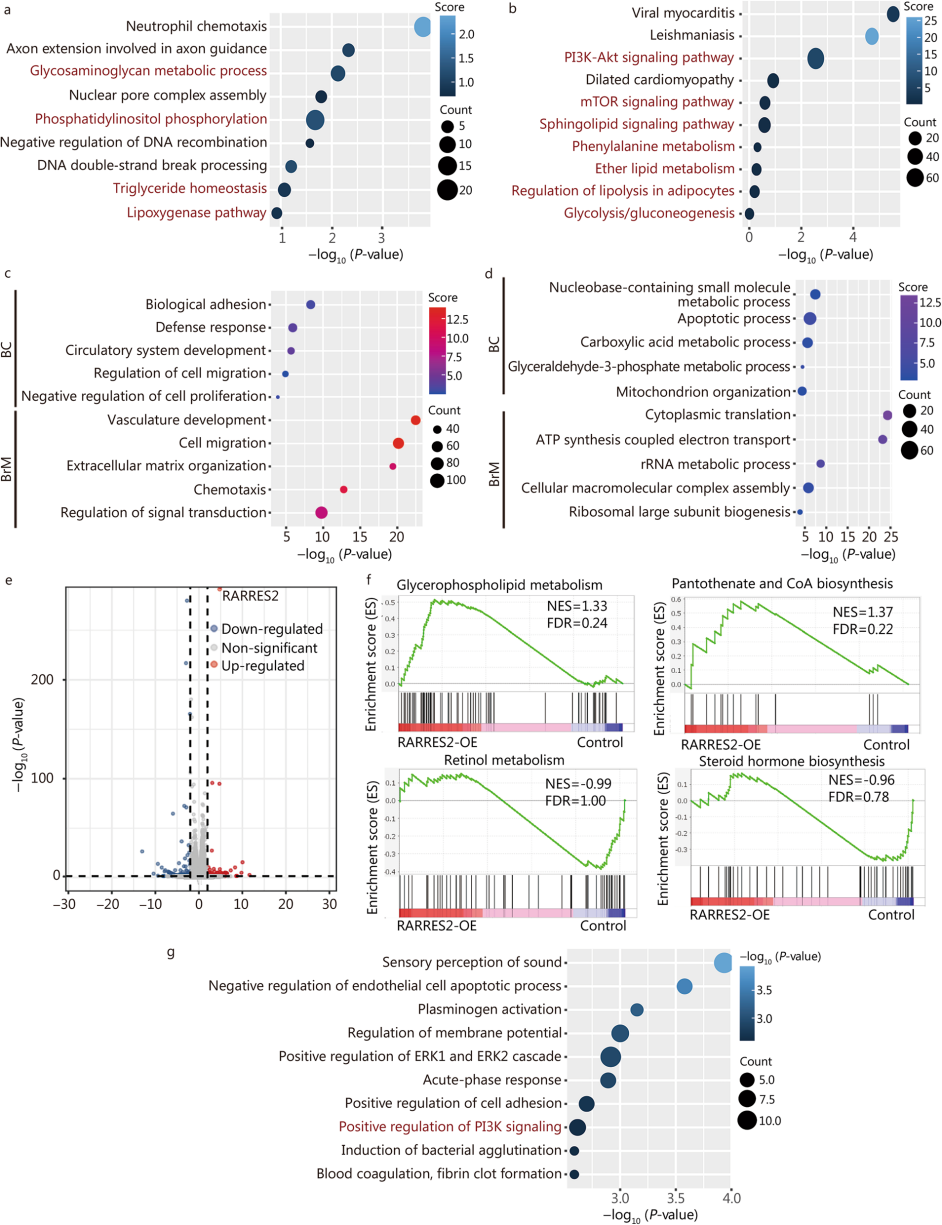
Enrichment analyses indicate RARRES2 regulates lipid metabolic reprogramming and PI3K signaling pathway in breast cancer. **a** and **b** RARRES2 co-expression genes in TCGA-BRCA were analyzed for enrichment in Gene Ontology (GO) biological processes (**a**) and Kyoto Encyclopedia of Genes and Genomes (KEGG) pathways (**b**). The size of each bubble represents the number of genes, while the color represents the enrichment score. **c** and **d** GO biological process enrichment analysis of RARRES2 positive (**c**) or negative (**d**) co-expression genes in scRNA-seq of primary and BrM cancer epithelial cells mentioned in Fig. 1d. **e** Volcano plot showing the differentially expressed genes between RARRES2-OE and control MDA-MB-231 cells. Genes expressed at significantly higher levels in RARRES2-OE group are shown in red; genes expressed at significantly lower levels, blue; genes expressed non-significantly differently in the two cell lines, gray. The dashed lines indicate the cut-off in log_2_ (FC) and log_10_ (*P-*value) for a gene to be considered differentially expressed. **f** Gene set enrichment analysis of the differentially expressed genes between RARRES2-OE and control MDA-MB-231 cells showed upregulation of glycerophospholipid metabolism and biosynthesis of pantothenate and CoA, as well as downregulation of retinol metabolism and steroid hormone biosynthesis. **g** Differentially expressed genes in panel (**e**) were analyzed for enrichment in GO biological processes. The size of each bubble represents the number of genes in the process, while the color represents the -log_10_ (*P*-value). RARRES2 retinoic acid receptor responder 2, PI3K phosphatidylinositol 3-kinase, FC fold change, BrM brain metastasis, scRNA-seq single-cell RNA-sequencing, RARRES2-OE RARRES2 overexpression, mTOR mammalian target of rapamycin, NES normalized enrichment score, FDR false discovery rate

To gain a deeper understanding of the molecular mechanisms underlying the modulation of BCBrM by RARRES2, we conducted an analysis to identify RARRES2 co-expression genes in TCGA-BRCA transcriptome sequencing data. Subsequently, Gene Ontology biological process and Kyoto Encyclopedia of Genes and Genomes enrichment analysis revealed that these genes were primarily involved in lipid metabolic pathways, including phosphatidylinositol phosphorylation, triglyceride homeostasis, lipoxygenase pathway, PI3K-Akt signaling pathway, and mTOR signaling pathway (Fig. 3a, b). These findings shed light on the potential involvement of these pathways in the RARRES2-mediated modulation of BCBrM.

Furthermore, we extended our investigation to identify RARRES2 co-expressed genes in the single-cell transcriptome data (Additional file 1: Fig. S1d) of breast cancer and BrM cancer epithelial cells respectively, and explored the biological processes regulated by RARRES2. Notably, genes exhibiting a positive correlation with RARRES2 were predominantly associated with tumor metastasis-related processes, including biological adhesion, cell migration and proliferation (Fig. 3c). On the other hand, genes showing a negative correlation with RARRES2 expression were primarily involved in material and acid metabolism (Fig. 3d). These findings provide further insights into the potential regulatory roles of RARRES2 in the biological processes associated with breast cancer metastasis.

To further investigate the molecular alterations associated with RARRES2 overexpression, we conducted transcriptomic sequencing in RARRES2-OE cells and control MDA-MB-231 cells. Differentially expressed genes [log_2_ (FC) > 1 or log_2_ (FC) <  − 1, *P* < 0.05, Fig. 3e] were shown to be enriched in metastatic biological processes, such as cell adhesion (Fig. 3g). In addition, functional enrichment analyses further highlighted lipid metabolic processes, including phospholipid efflux. Moreover, gene-set enrichment analysis based on the Kyoto Encyclopedia of Genes and Genomes database indicated upregulation of glycerophospholipid metabolism and biosynthesis of pantothenate and CoA, while retinol metabolism and steroid hormone biosynthesis were downregulated in RARRES2-OE group (Fig. 3f). Notably, pathway analyses of differentially expressed genes also identified PI3K signaling pathway (Fig. 3g). These findings provide insights into the molecular changes associated with RARRES2 overexpression, particularly highlighting alterations in metastatic processes, lipid metabolism, and the PI3K signaling pathway.

By integrating the findings from our analyses of public databases, single-cell transcriptome sequencing data, and transcriptomic profiling of RARRES2-OE cells, we have formulated a hypothesis that RARRES2 may play a role in the regulation of lipid metabolic reprogramming through PI3K signaling pathway to mediate BCBrM.

### RARRES2 alters lipid metabolism in MDA-MB-231 cells

**Fig. 4:**
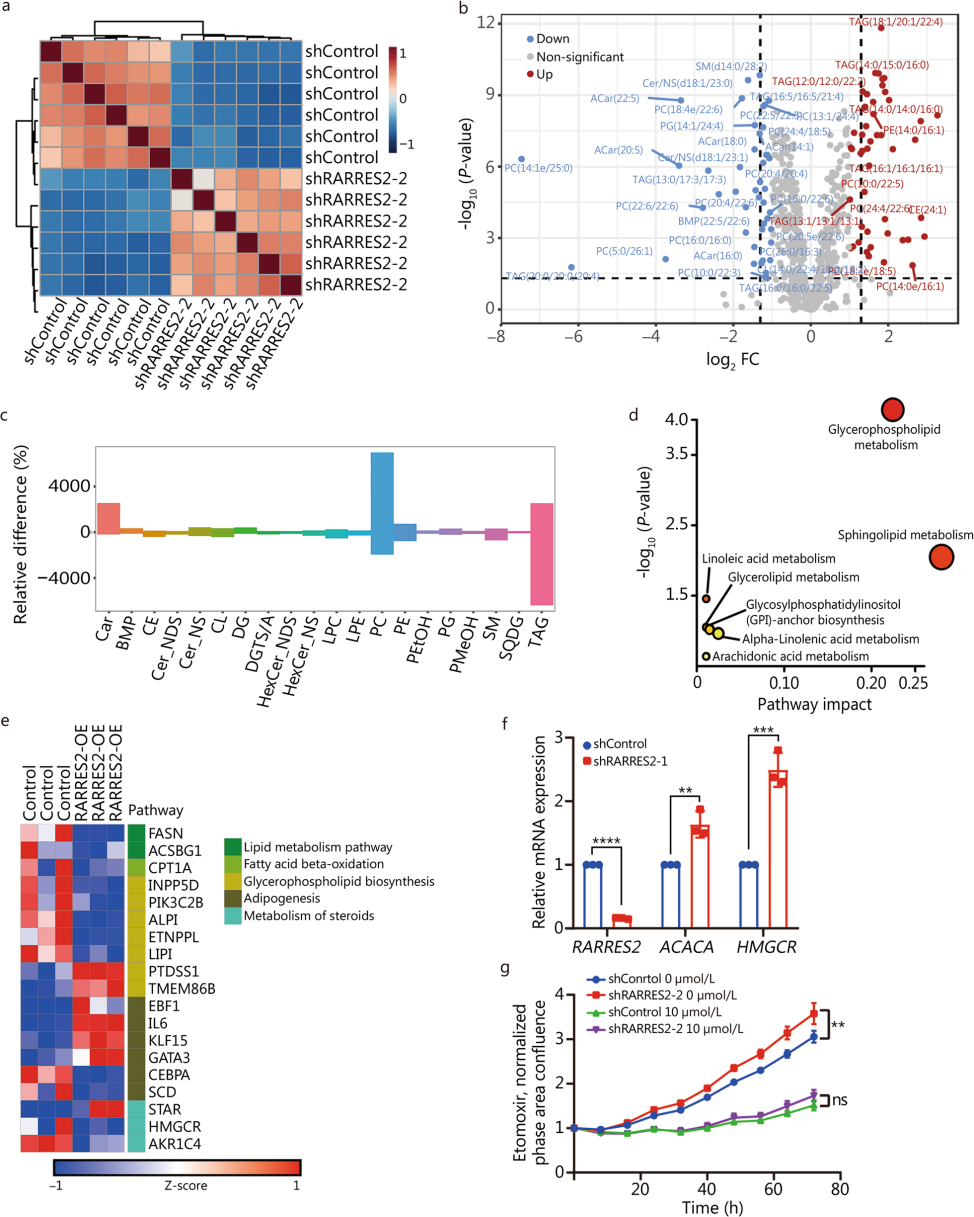
RARRES2 regulates lipid metabolism in MDA-MB-231 cells. **a** Correlation heatmap shows the lipid profiles between shRARRES2-2 and shControl MDA-MB-231 cells detected by untargeted lipidomic analysis. **b** Volcano plot showing the differentially expressed lipid metabolites in shRARRES2 in comparison to shControl samples. Blue and red circles represent lipid species that were significantly downregulated and upregulated, respectively, in shRARRES2 cells. **c** Relative differences in the content of lipid species between shRARRES2 and shControl MDA-MB-231 cells. **d** Metabolic pathways that were significantly upregulated in shRARRES2 MDA-MB-231 cells, based on our lipidomic profiling and the KEGG human metabolome database. **e** Heatmap of lipid metabolism genes differentially expressed between RARRES2-OE and control MDA-MB-231 cells. Each column in the matrix corresponds to a sample. Blue squares indicate genes downregulated in RARRES2-OE cells; red squares indicate genes upregulated in RARRES2-OE cells. **f** mRNA levels of ACACA and HMGCR in shRARRES2-1 and shControl MDA-MB-231 cells were examined by RT-qPCR. Levels of mRNA were normalized to those of GAPDH. ^**^P = 0.006, ^***^P = 0.0007, ^****^P < 0.0001 (two-tailed unpaired-samples *t* test). **g** Viability of shRARRES2-2 and control MDA-MB-231 cells treated with 10 μmol/L etomoxir. The differences were tested for their significance using two-way ANOVA with Geisser-Greenhouse correction: ^**^P = 0.0079. RARRES2 retinoic acid receptor responder 2, KEGG Kyoto Encyclopedia of Genes and Genomes, ACACA acetyl-CoA carboxylase alpha, HMGCR hydroxymethylglutaryl-CoA reductase, RT-qPCR real-time quantitative polymerase chain reaction, Car acylcarnitine, BMP bismonoacylglycerophosphate, CE cholesteryl ester, Cer_NDS ceramide non-hydroxyfatty acid-dihydrosphingosine, Cer_NS ceramide non-hydroxyfatty acid-sphingosine, CL cardiolipin, DG diacylglycerol, DGTS/A diacylglyceryl trimethylhomoserine/diacylglyceryl hydroxymethyl-N,N,N-trimethyl-β-alanine, HexCer_NDS hexosylceramide non-hydroxyfatty acid-sphingosine, HexCer_NS hexosylceramide non-hydroxyfatty acid-dihydrosphingosine, LPC lysophophatidylcholine, LPE lysophosphatidylethanolamine, PC phosphatidylcholine, PE phosphatidylethanolamine, PEtOH phosphatidylethanol, PG phosphatidylglycerol, PMeOH phosphatidylmethanol, SM sphingomyelin, SQDG sulfoquinovosyl diacylglycerol, TAG triacylglycerol, RARRES2-OE RARRES2 overexpression, ns non-significant

To investigate the role of RARRES2 in regulating lipid metabolism in BCBrM, we conducted untargeted lipidomic profiling in RARRES2-KD and its control MDA-MB-231 cells, and observed remarkable alterations in lipid metabolites (Fig. 4a, b). Specifically, we noted an increase in the levels of phosphatidylcholine (PC) and phosphatidylethanolamine (PE), which are crucial components of the glycerophospholipid family (Fig. 4c). In contrast, there was a decrease in the levels of TAGs (Fig. 4c). Pathway analysis of differentially expressed lipids revealed a significant enrichment of glycerophospholipid metabolism, sphingolipid metabolism, and glycerolipid metabolism (Fig. 4d). Furthermore, RNA-sequencing of RARRES2-OE and its control MDA-MB-231 cells revealed that RARRES2 regulates the expression of genes involved in the glycerophospholipid metabolism, fatty acid synthesis, and cholesterol synthesis, such as FASN, stearoyl-CoA desaturase (SCD), HMGCR, carnitine palmitoyltransferase 1A (CPT1A), and phosphatidylserine synthase 1 (PTDSS1) expression etc. (Fig. 4e). Additionally, RT-qPCR also confirmed the upregulated expression of ACACA and HMGCR following RARRES2 knockdown (*P *= 0.006, *P* = 0.0007, *P * < 0.0001, Fig. 4f). These findings strongly indicate that decreased expression of RARRES2 is closely associated with dysregulated lipid metabolism in MDA-MB-231 cell line, suggesting a potential role for RARRES2 in modulating lipid metabolism in BCBrM. Glycerophospholipids, particularly PC, are the predominant structural lipid components of cellular membranes. PC accounts for over 50% of all membrane phospholipids and is primarily enriched in the outer leaflet of neurons and glial cells. It serves as both a structural lipid and a signaling lipid, supporting normal brain function. In contrast, TAGs, the common storage form of fatty acids, are considerably less abundant in the brain compared to other tissues [8]. This disparity suggests that the brain accumulates specialized lipids to support neural activity, rather than relying on TAGs. As for fatty acid metabolism, previous study has indicated that non-brain-metastatic cells exhibit a fatty acid oxidation signature, whereas fatty acid synthesis is necessary for BCBrM [9]. Based on these observations, it is reasonable to propose that RARRES2-downregulated breast cancer cells decreased cellular level of TAGs to adapt to the low-TAG microenvironment of the brain. Simultaneously, they increase cellular levels of PC to facilitate cancer cell growth in the brain, aligning with the seed-and-soil hypothesis [21].

To investigate whether interference with RARRES2-mediated lipid metabolic reprogramming affects the malignant phenotype of breast cancer cells, we conducted experiments using etomoxir, a CPT1 inhibitor, on MDA-MB-231 cell line with altered RARRES2 expression. Interestingly, we observed that in RARRES2 knockdown cells, the inhibition of CPT1 significantly reversed cell proliferation compared with control cells (*P* = 0.0079, Fig. 4g). Collectively, the combination of genetic, metabolic, transcriptomic, and functional evidence provides compelling support for the role of RARRES2 in regulating lipid metabolism.

### RARRES2 negatively regulates PTEN-mTOR-SREBP1 signaling pathway

Lipid metabolic reprogramming is tightly regulated by various metabolic pathways involved in the synthesis and degradation metabolites. Notably, the PI3K/mTOR axes and their downstream SREBP signaling [22], emerged as key regulatory hubs in lipid metabolism.

**Fig. 5:**
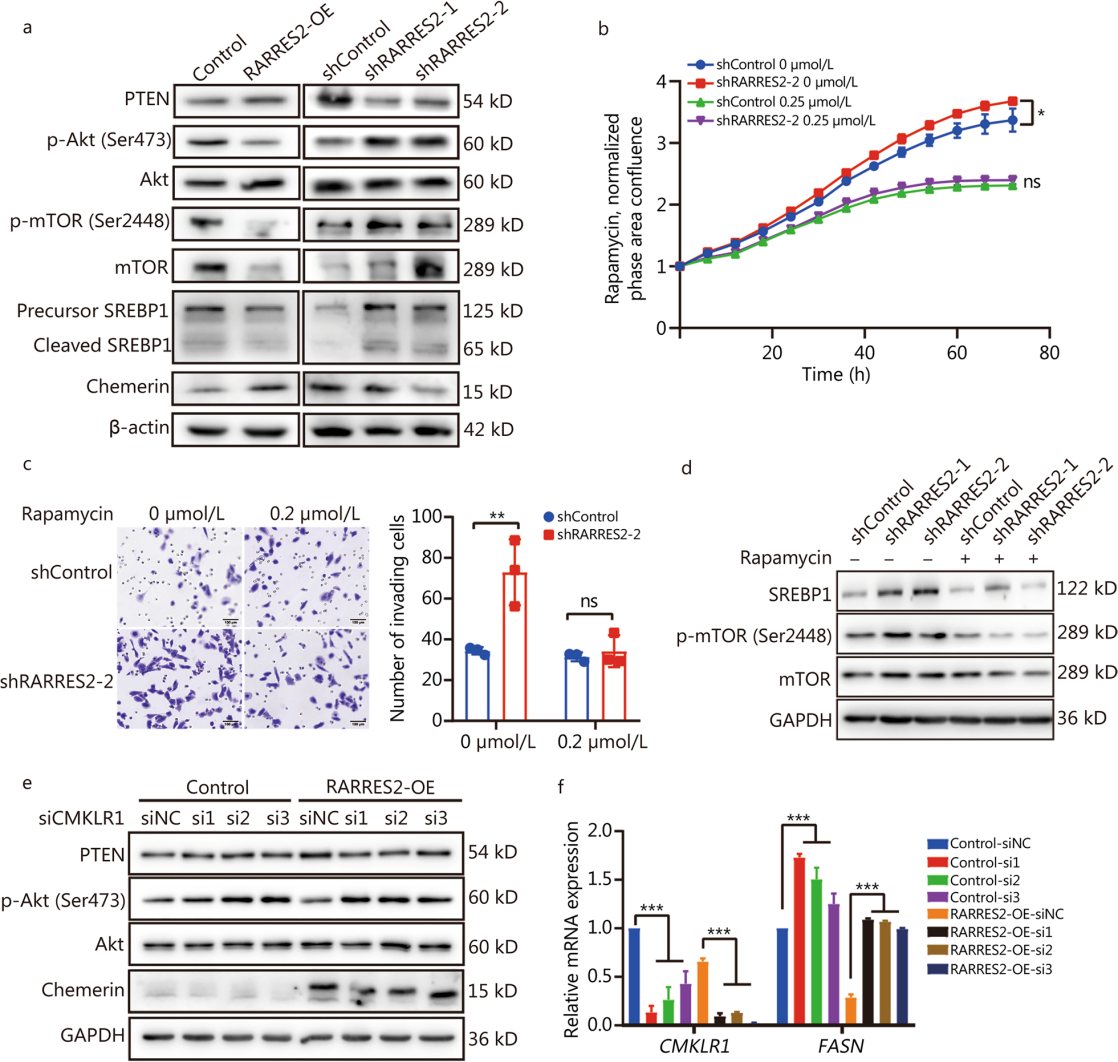
RARRES2 negatively regulates the PTEN-mTOR-SREBP1 axis.** a** Western blotting analysis of the levels of PTEN, Akt, p-Akt (Ser473), mTOR, p-mTOR (Ser2448), SREBP1 and Chemerin in RARRES2-OE, shRARRES2 and control MDA-MB-231 cells. **b** Viability of shRARRES2-2 and control MDA-MB-231 cells treated with 0.25 μmol/L rapamycin. Two-way ANOVA with Geisser-Greenhouse correction: ^*^P = 0.0017. **c** Rapamycin treatment (0.2 μmol/L) rescues cell invasion in shRARRES2-2 MDA-MB-231 cells (Scale bar = 100 μm). Two-way ANOVA with Sidak’s multiple comparisons test: ^**^P = 0.0133. **d** Western blotting analysis of the levels of mTOR, p-mTOR (Ser2448) and SREBP1 in shRARRES2 and control MDA-MB-231 cells treated with 0.2 μmol/L rapamycin. **e** Western blotting of total lysates of RARRES2-OE and control MDA-MB-231 cells, which were transfected with short interfering RNA against CMKLR1 (si1 – 3) or a negative-control RNA (siNC). **f** mRNA levels of CMKLR1 and FASN in the cultures described in panel (**e**). Levels of mRNAs were normalized to those of GAPDH. ^***^P < 0.001 (one-way ANOVA followed by Dunnett’s multiple-comparisons test). RARRES2 retinoic acid receptor responder 2, mTOR mammalian target of rapamycin, SREBP sterol regulatory element-binding protein, CMKLR1 chemokine-like receptor-1, FASN fatty acid synthase, RARRES2-OE RARRES2 overexpression, ns non-significant

Recent evidence has highlighted the significance of PIK3CA mutation as the primary mutation associated with BCBrM, and the essential role of SREBP1 in promoting breast cancer cell proliferation in the brain [8]. A study disrupting lipid metabolism in brain-tropic TNBC cells (HCC1806) through SREBP1 knockout has demonstrated a significant halt in BCBrM [8]. Drawing from these findings and our functional enrichment analyses (Fig. 3b, g), we propose that RARRES2 may modulate lipid metabolic reprogramming through the mTOR-SREBP1 signaling pathway. In line with this hypothesis, the protein levels of p-Akt, mTOR, p-mTOR, SREBP1/cleaved SREBP1 varied inversely with RARRES2-KD or RARRES2-OE (Fig. 5a), indicating a negative regulatory role of RARRES2 on the mTOR-SREBP1 signaling pathway in MDA-MB-231 cells. Furthermore, we corroborated the association between RARRES2 and SREBF1 in an independent patient cohort of 22 BCBrM cases [20], and found a negative correlation between RARRES2 expression and SREBF1 (Additional file 1: Fig. S3). Another critical player in this context is PTEN, a tumor suppressor frequently lost in TNBC and associated with PI3K pathway activation [23]. The percentage of PTEN loss/mutation has been reported to be as high as 35–40% in TNBC [24]. Moreover, PTEN was within the top 5 genes with genomic alterations in a genomic landscape of BCBrM [25]. Previous studies have reported that RARRES2 regulates the PI3K-mTOR axis via PTEN [13, 26], and we observed that the protein level of PTEN varied directly with RARRES2 (Fig. 5a).

To investigate whether the pro-oncogenic effects of RARRES2 deficiency can be reversed by inhibiting the mTOR pathway, we treated RARRES2-KD and its control MDA-MB-231 cells with the mTOR inhibitor rapamycin. The results demonstrated that rapamycin treatment significantly reversed the increased proliferation and invasion observed in RARRES2-KD cells (*P *= 0.0017, *P* = 0.0133, Fig. 5b, c). Furthermore, Western blotting analysis revealed that rapamycin treatment reversed the elevated levels of p-mTOR and SREBP1 expression induced by RARRES2 knockdown (Fig. 5d).

CMKLR1 is the primary receptor through which RARRES2 exerts its biological functions [27]. Therefore, we further investigated the role of CMKLR1 in RARRES2-mediated lipid metabolic reprogramming and its downstream signaling. Knockdown of CMKLR1 abolished the significant increase in PTEN expression and the decrease in p-Akt expression observed in RARRES2-OE cells (Fig. 5e). Additionally, CMKLR1 knockdown resulted in an upregulation of FASN expression at the mRNA level in both RARRES2-OE and control cells (*P *< 0.001, Fig. 5f). These findings collectively suggest that RARRES2 regulates the PTEN-mTOR-SREBP1 axis, which in turn regulates lipid metabolism and BCBrM in TNBC.

## Discussion

Little is known about how brain-tropic breast cancer cells adapt to the unique brain microenvironment. One distinct feature of the brain microenvironment is the enrichment of lipids, which determines the properties and organization of neurons and glial cell membranes, as well as engages in intracellular and transcellular signaling to maintain normal brain function. Recent evidence suggests an important role of lipid metabolic reprogramming in BCBrM [8, 9]. However, the mechanisms are far from being fully uncovered and no therapeutic targets have been identified. In this study, we identified RARRES2, a multifunctional adipokine and chemokine, as an essential link between BCBrM and lipid metabolic reprogramming. We uncovered the role of RARRES2 as a metabolic mediator that shifts intracellular lipid contents of brain metastatic potential TNBC cells through PTEN-mTOR-SREBP1 signaling pathway to facilitate cell survival in the brain microenvironment. We provided evidence of RARRES2-mediated lipid metabolic reprogramming as a previously unidentified mechanism in the development of BCBrM.

While previous studies of RARRES2 mainly focused on autoimmune diseases and obesity [11, 12], our study reveals a role of RARRES2 in lipid metabolism in BCBrM. We observed an upregulation of glycerophospholipids and a downregulation of TAGs following RARRES2 knockdown. From the physiological point of view, the brain has a unique microenvironment. Lipids constitute approximately 50% of the brain, making brain the “fattiest” organ in the human body. The lipid composition of the brain is dominated by phospholipids, including glycerophospholipids and sphingolipids, followed by gangliosides and cholesterol, while the level of TAGs is low [28]. Consistent with our findings, Jin et al. [8] recently reported that highly brain-metastatic cells displayed increased levels of glycerophospholipid and cholesterol species while the levels of TAGs were decreased. Zhu et al. [29] also observed a passive TAG accumulation after RARRES2-OE in hepatic lipid metabolism. One possible explanation for this lipid metabolism reprogramming is that breast cancer cells need to adapt to this low-TAG, high-glycerophospholipid microenvironment and obtain glycerophospholipid via de novo synthesis or from other routes to survive in the brain. Jin et al. [8] also identified that perturbation of lipid metabolism through knocking out SREBP1 in brain-tropic breast cancer cells, including TNBC cells, halted BCBrM. In fact, glycerophospholipids itself are closely associated with tumor migration and metastasis. One form of glycerophospholipids, the phosphatidylinositides were found to serve as membrane trafficking regulators in cancer cells, and phosphatidylinositol 3,4,5-trisphosphate and phosphatidylinositol 4,5-bisphosphate regulated cellular processes by impacting actin dynamics, thus causing alterations in cellular metastatic capacity [30, 31]. Although the mechanisms linking lipid metabolism alterations and BrM require further investigation, our data reveal a role of RARRES2 in this process and raise the potential therapeutic opportunity of targeting RARRES2 in BCBrM.

Our study also showed that RARRES2 regulated lipid metabolism through PTEN-mTOR-SREBP1 signaling pathway. A few studies have also reported the positive correlation between RARRES2 and PTEN via its G protein-coupled receptor CMKLR1 [13, 26]. CMKLR1 is the predominant receptor of RARRES2, and is found in microglia, ependymal cells, and tanycytes in the mouse brain [32]. Previous studies have also demonstrated a negative regulation of RARRES2 and PI3K pathway, which were in accordance with our findings [13, 33]. PI3K is not only an important downstream mediator of human epidermal growth factor receptor 2 but also reported to be associated with BCBrM [34]. Moreover, PI3K pathway mutation showed the top correlation associated with BCBrM in brain-metastatic cell lines. Furthermore, PI3K pathway plays an important role in lipid anabolism by activating the expression of several genes involved in cholesterol and fatty acid biosynthesis [35]. SREBPs, downstream of the PI3K/mTOR axis, are essential transcription factors mediating lipid synthesis [36]. SREBP1 was found to be selectively required for the growth of brain-metastatic cells compared with cells without brain-metastatic potential. This metastatic necessity was specific to the brain but not to other organs [8]. Our results demonstrated that RARRES2 negatively regulated the expression of mTOR-SREBP1, which not only revealed the detailed mechanism of RARRES2 in regulating lipid metabolism, but more importantly, reinforced the specific association between RARRES2 and BrM. Of note, we mainly focused on TNBC in this study, and the association between RARRES2 and BCBrM in other breast cancer subtypes needs further investigation.

## Conclusions

In summary, our current study focused on BrM, the clinical dilemma of breast cancer, and identified the specific association between RARRES2 and BCBrM. We provided evidence that lipid metabolic reprogramming mediated by RARRES2 is of essential importance for brain metastatic formation in TNBC. Our results not only add new insights to the current understanding of lipid metabolism and breast cancer BrM, but also suggest potential therapeutic targeting of RARRES2 as an intervention for this life-threatening disease.

### Supplementary Information

**Additional file 1: Fig. S1** BCBrM tumor cluster showed specific gene signatures compared to primary TNBC tumor cells. **Fig. S2** Expression of RARRES2 in different tissues, based on data from the Genotype-Tissue Expression (GTEx) project. **Fig. S3** Correlation of RARRES2 mRNA expression with that of SREBF1 in 22 breast cancer brain metastasis tissues.

## Data Availability

The data used to support the findings of this study are available from the corresponding authors upon reasonable request.

## Abbreviations

ACACA: Acetyl-CoA carboxylase alpha
BCBrM: Breast cancer brain metastasis
BrM: Brain metastasis
CMKLR1: Chemokine-like receptor-1
CPT1: Carnitine palmitoyltransferase 1
FASN: Fatty acid synthase
FBS: Fetal bovine serum
HMGCR: Hydroxymethylglutaryl-CoA reductase
IHC: Immunohistochemistry
KD: Knockdown
mTOR: Mammalian target of rapamycin
OE: Overexpression
PC: Phosphatidylcholine
PBS: Phosphate-buffered saline
PE: Phosphatidylethanolamine
PI: Phosphatidylinositides
PI3K: Phosphatidylinositol 3-kinase
PTDSS1: Phosphatidylserine synthase 1
SCD: Stearoyl-CoA desaturase
scRNA-seq: Single-cell RNA-sequencing
SREBP: Sterol regulatory element-binding protein
TAG: Triacylglycerol
TNBC: Triple negative breast cancer
