# Supplementary material for: RARRES2 regulates lipid metabolic reprogramming to mediate the development of brain metastasis in triple negative breast cancer
Source: Mil Med Res. 2023 Jul 25;10:34. doi: 10.1186/s40779-023-00470-y (PMC10369725; doi:10.1186/s40779-023-00470-y)
Supplement: Supplementary file 1 — Additional file 1: Fig. S1 BCBrM tumor cluster showed specific gene signatures compared to primary TNBC tumor cells. Fig. S2 Expression of RARRES2 in different tissues, based on data from the Genotype-Tissue Expression (GTEx) project. Fig. S3 Correlation of RARRES2 mRNA expression with that of SREBF1 in 22 breast cancer brain metastasis tissues. [file 40779_2023_470_MOESM1_ESM.pdf]

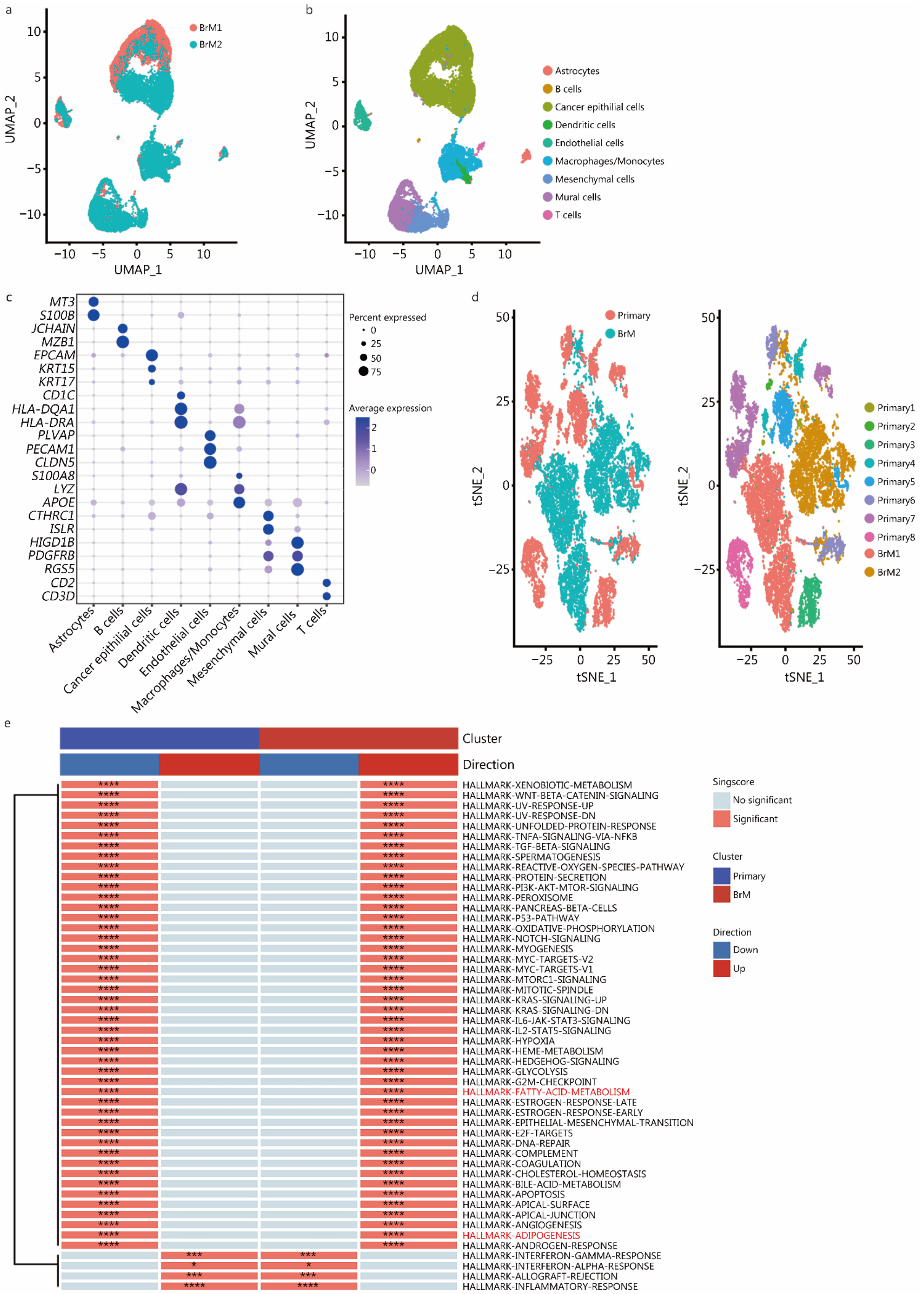

Fig. S1 BCBrM tumor cluster showed specific gene signatures compared to primary TNBC tumor cells. **a** and **b** UMAP visualization of scRNA-seq profiles of 2 TNBC BrM tissues (BrM1 from our patients, BrM2 from

GSE186344) displayed the annotation for 9 cell subclusters. **c** Dot plots indicated expression of canonical annotation marker genes. **d** A tSNE visualization of scRNA-seq profiles of human TNBC (Primary, 8 samples from GSE176078) and BrM (2 samples above) cancer epithelial cells. **e** Singscore of significantly different Hallmark pathways in primary and BrM cancers. $^{*}P < 0.05$, $^{***}P < 0.001$, $^{****}P < 0.0001$. BCBrM breast cancer brain metastasis, TNBC triple negative breast cancer, UMAP uniform manifold approximation and projection, scRNA-seq single-cell RNA-sequencing, BrM brain metastasis

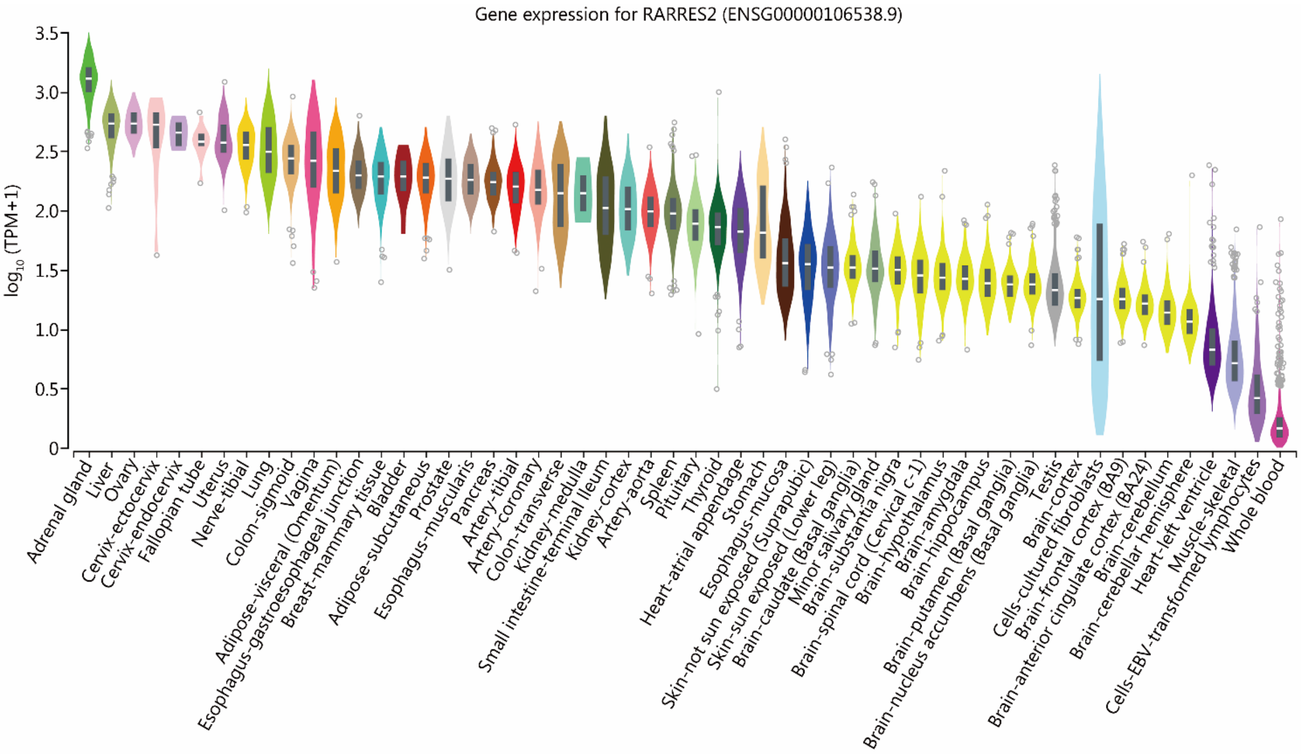

Fig. S2 Expression of RARRES2 in different tissues, based on data from the Genotype-Tissue Expression (GTEx) project. RARRES2 retinoic acid receptor responder 2, TPM transcripts per million

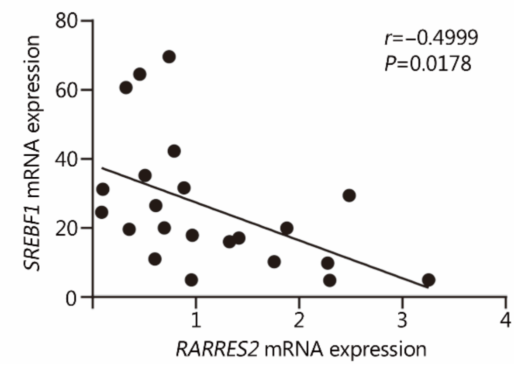

Fig. S3 Correlation of *RARRES2* mRNA expression with that of *SREBF1* in 22 breast cancer brain metastasis tissues. RARRES2 retinoic acid receptor responder 2, SREBF1 sterol regulatory element binding transcription factor 1
